# Multi-method, longitudinal study on behavioral, emotional, and developmental factors of children in residential care in Lower Austria: A study protocol

**DOI:** 10.1371/journal.pone.0352579

**Published:** 2026-07-09

**Authors:** Sarah Buttler-Stöbich, Rachel Dale, Katja Haider, Doris Mayerhofer, Raphaela Kohout, Ilona Horwath, Christoph Pieh

**Affiliations:** Center for Child and Youth Welfare, Department for Psychosomatic Medicine and Psychotherapy, University for Continuing Education Krems, Krems an der Donau, Austria; PNG National Research Institute, PAPUA NEW GUINEA

## Abstract

**Background:**

In Austria, there is a significant research gap concerning the quality of out-of-home care (OoHC) provisions and their capacity to promote children’s well-being and development. Given the intrusive nature of such interventions, thorough evaluation is essential. This study protocol outlines a multi-method, long-term, and multi-perspective study that investigates: 1) the circumstances leading to full-time residential care placements; 2) the development of behavioral, emotional, and social factors over time; 3) key factors influencing children’s outcomes in residential care 4) the structure and everyday reality of residential care in Lower Austria, a federal state of Austria and 5) that generates knowledge that will inform improvements in child welfare practice and policy.

**Methods:**

The first three research questions will be addressed through a longitudinal quantitative study involving children and adolescents (aged 11–21 years), their parents, and professionals working in residential care settings. Standardized surveys will be administered at multiple time points to track developmental changes and identify influencing factors.

In addition, to gain a deeper understanding of the implicit mechanisms, and everyday practices of residential care, we plan to implement accompanying qualitative methods adapted to the particular circumstances of the field of research. Finally, a communicative validation process will be used to discuss and reflect on preliminary findings with professionals in the field. This step ensures the relevance of the results and supports the development of practice-oriented knowledge for quality improvement and safeguarding in child welfare services.

**Discussion:**

This first-of-its-kind study in Austria combines quantitative and qualitative methods to generate evidence for child welfare practice and policy. An innovative element is the potential development of a standardized documentation tool to support research and quality assurance. Challenges include the field’s complexity, participant recruitment, and risk of participation fatigue, which require close collaboration with practitioners and flexible survey strategies to ensure relevant results.

## Introduction

In Austria 13,050 children lived in out-of-home care in 2024 [1]. This form of intervention is typically employed when a child’s safety and well-being are endangered within their family environment, and when less invasive child protection measures have failed [2]. Among these arrangements, residential care, which is primarily organized in the form of small group homes staffed by professional pedagogues, remains the predominant placement type, accounting for 61.7% of all out-of-home placements, compared to 38.3% in foster care [1].

However, placement in care represents one of the most intrusive interventions available within the child welfare system and is not without risks. Placements are associated with complex developmental, emotional, and social consequences that must be carefully considered [3,4] but seem necessary in cases of severe neglect, maltreatment, or abuse.

There is currently a significant research gap in Austria regarding the benefits of various forms of care for children who cannot live with their families [5]. Internationally, findings are similarly inconclusive, with some studies reporting improvements and others indicating deteriorations in children’s outcomes following placement in OoHC [3,6,7]. For instance, based on a systematic review of quantitative research that compared developmental health or wellbeing outcomes for maltreated children placed in out-of-home care with those cared for in their home, Maclean et al. [3] found that out of 40 studies, 29 were consistent with no benefit or harm of out-of-home placement, seven with harm and four with benefit; three studies found no evidence of significant difference or found worse outcomes for out-of-home cared children. In addition, cohort studies suggested limited evidence of improved outcomes as well as some evidence for worse outcomes. In contrast, [6] investigated the mental health development of 8–17-year-olds in out-of-home care in Spain and found that after two years, 29.9% showed sustained mental health, 26% meaningful improvement, 23.5% meaningful deterioration and 20.5% no meaningful change. The authors conclude that it is crucial to establish protocols and systematic detection tools to assess the mental health of children and adolescents in out-of-home care, which is also true for Austria and forms the major aim of our research project. [7] drew on the Child-Behaviour Checklist (CBCL) and the Assessment Checklist for Children (ACC) and Adolescents (ACA) to measure the seven-to nine-year stability of (carer-reported) scores for children in long-term foster or kinship care. While most children showed either sustained mental health or meaningful improvements, the scores for the aggregate sample did not change. As a uniform, population-wide effect of out-of-home placement cannot be assumed – rather, children’s mental health development follows distinct trajectories – research should focus on questions of systemic and interpersonal characteristics of care that sustain their psychological development, of characteristics that may be developmentally harmful and on the question for which children care may be therapeutic, or not.

However, the reported studies [3,6,7] focus on mental health, with different analytical lenses and with different methodological approaches. Commonly, a key methodological challenge is the high risk of bias, as children entering OoHC often come from significantly more disadvantaged backgrounds and face a higher likelihood of poor outcomes than other maltreated children. This makes it difficult to determine whether negative outcomes are caused by the care itself or by the complex histories of the children involved [3].

Moreover, the measurement of outcomes in social work is controversial and methodologically challenging [8]. Critics argue that the non-operationalizable nature of social work processes, the lack of congruence between client and professional goals, and the diversity of organizational approaches across federal regions in Austria all contribute to the difficulty in establishing clear indicators of impact [5,8]. This complexity is compounded by the fact that “residential care” is rarely clearly defined in existing studies, and the actual implementation of interventions remains insufficiently described or lacks transparency [9,10].

Existing evaluations have often focused narrowly on “outcomes” or aggregated social impacts, without sufficiently examining the mechanisms and contextual factors that shape these outcomes [11]. However, such a limited perspective overlooks the fact that in child and youth welfare, quality cannot be assessed by results alone. The nature of the interventions requires professionals to not only act with expertise but also to critically reflect on the impact of their actions. Quality, therefore, must be understood as a multidimensional construct that includes structural conditions (such as staffing, funding, and organizational support), process quality (such as transparency, participatory practices, and respectful interaction), and the ability to respond to diverse and sometimes conflicting expectations from children, parents, and society. A comprehensive evaluation of youth welfare services must therefore move beyond outcome metrics and consider the full complexity of professional practice and lived experiences [12]. Additionally, what is defined as a “successful” outcome for children who have experienced severe adversity and trauma, often with long-lasting effects, must be considered with particular care and sensitivity [7].

In light of these challenges, a more nuanced and context-sensitive approach is required to assess the quality and effectiveness of residential out-of-home care. In that regard, the present longitudinal study represents the first attempt to gain a deeper understanding of residential care in the federal state of Lower Austria. To support this effort, a dedicated research center was established at the University for Continuing Education Krems, tasked with accompanying and scientifically evaluating the work of the regional child and youth welfare services. In close collaboration with field experts and practitioners, we developed a research design that aims to investigate the developmental pathways of children and adolescents placed in residential care. Special attention is given to identifying protective and risk factors that influence long-term outcomes. By adopting this comprehensive perspective, the study seeks to generate empirically grounded insights into which care environments and contextual factors most effectively support positive development. The findings are intended to inform and enhance evidence-based practices within the Austrian child welfare system.

### Objectives of the research study

This research aims to address gaps in knowledge regarding residential care within the child welfare system in Lower Austria. Its primary objectives are to 1) analyze the conditions under which children and their families become recipients of full-time care services, 2) examine how behavioral, emotional, and developmental variables evolve during residential care, 3) identify key factors which influence children’s and adolescent’s development in this setting, and 4) enhance understanding to inform improvements in child and youth welfare services.

As conditions, we consider initial factors that predate the OoHC measurement but may nevertheless have an impact on its course, specifically resources and burdens on children and young people or the family system; reasons for and duration of child and youth welfare involvement in a case; demographic factors such as age and gender [13,14].

## Materials and methods

### Overview of the research design

The study is conducted in close collaboration with child welfare experts to ensure practical relevance and is grounded in thorough literature research. It employs a multi-perspective, longitudinal approach, incorporating the perspectives of children and adolescents, parents (or other close family members), and professional caregivers responsible for daily care and support of children and adolescents in out-of-home settings. To address objectives 1 and 2 longitudinal data on behavioral, emotional, and developmental variables are collected through standardized surveys. For objective 3, a qualitative part of the study provides in-depth insights into the structures and processes of residential care in Lower Austria. Communicative validation with practitioners ensures that the interpretation of findings is grounded in professional expertise and supports the applicability of results (objective 4).

### Long-term quantitative study design

The longitudinal study is based on a model that identifies key variables across different stages of the child welfare process, including pre-care factors that lead to out-of-home placement, in-care factors that influence the child’s experience in residential care, and outcome factors that reflect short- and mid-term developmental changes. Each group of interest, including children and adolescents, parents (or other close family members), and professional caregivers, will receive tailored surveys at different time points.

In the initial phase, a cross-sectional convenience sample will be recruited, consisting of all children and adolescents currently residing in residential care facilities in Lower Austria who meet the inclusion criteria (age and sufficient German reading skills) and provide informed consent to participate, regardless of the duration of their stay in care. In addition to the children themselves, their professional caregivers and, where applicable, their parents (or other close family members) will also be invited to participate in the study to provide complementary perspectives. These participants will also be invited to take part in repeated follow-up assessments at regular intervals, enabling a longitudinal component.

In subsequent phases, the study will adopt a prospective cohort design. All newly admitted children entering residential care during the recruitment period will be enrolled at baseline (i.e., upon admission) and followed over time through repeated assessments. This prospective component will enable the examination of intra-individual changes in psychosocial, emotional, and developmental outcomes across the duration of their placement.

The combination of a cross-sectional baseline cohort with a prospective longitudinal recruitment strategy allows for both descriptive and inferential analyses across multiple time points. This design supports the investigation of both short- and long-term effects of residential care placement on children.

### Recruitment and sample size

The survey will be conducted online via the ESMira app [15], and Limesurvey [16] and disseminated through all residential care institutions in Lower Austria via the regional child and youth welfare services. Professionals will receive access to the survey through email or a physical newsletter containing a QR code linking to all three target group surveys. These professionals then invite eligible children and adolescents (aged 11–21 with sufficient German reading skills) and their parents (or other close family members) to participate.

The feasibility of large-scale data collection is currently unknown for this sample. In 2024, 2720 children and adolescents between 0–21 were accommodated in residential care in Lower Austria and more than half of the individuals fall within the eligible age range for participation in the study [1]. The participation rate cannot be predicted, as it will largely depend on the resources and motivation of the professional caregivers and their institutions. Recruiting parents (or other close family members) may be particularly challenging, as they are not in direct contact with professional caregivers.

Furthermore, the existing literature does not allow for a reliable estimation of the dropout rate. Comparable studies have reported dropout rates ranging from 24% to 76% over observation periods of 2–10 years [6,7,17,18]. To ensure the basic analyses can be conducted, i.e., the influence of demographic and care-related variables (age, gender, migration background, time in care system, reason for entering care system) on behavioral and emotional outcomes, a minimum sample size of N = 50 participants will be required at the initial phase. This corresponds to the standard rule of 10 data points per model factor for linear regression analyses [19]. The goal is to achieve development of a standardized documentation tool for the child and youth welfare service in Lower Austria, which would allow for more complex analyses at later time points.

### Procedure

To evaluate feasibility and usability of our research tools, cognitive pre-tests [20] were conducted in July-September 2025 with 9 participants (5 children & adolescents, 1 parent, 3 professionals). During the pre-tests, the questions were analyzed together with the various target groups to ensure they were comprehensible, and clear. Participants were also invited to review the questions, and suggest any additions, or improvements they considered appropriate. Based on this feedback, the surveys were revised. The official survey launch is scheduled for autumn 2025 and will remain open-ended. From that point, the survey will be conducted twice a year (May and October) during the first year, and subsequently once a year to enhance the likelihood of long-term participation.

The number of items per survey varies depending on the recipient group and time point. Table 1 outlines the key variables, the standardized instruments used, the recipient groups, and the time points for data collection. By combining standardized measures with context-specific items, the instruments allow for a comprehensive analysis of how personal, organizational, and systemic elements interact in everyday practice.

**Table 1:** Instruments.

Factors	Scale	Informant	Assessment Point		
			**T0**	T1*	**T2**
Demographics	Custom items	- Professionals - Children and adolescents from the age of 11–21 - Parents	x		
Professional background	Custom items	- Professionals	x	x	
Work situation	Custom items	- Professionals	x	x	
Child & family specific information	Custom items	- Professionals - Parents	x	x	
Family Characteristics	Custom items based on an evaluation by Kapella et al. [21]	- Professionals	x	x	x
Behavioral & Emotional Factors	CBCL/6–18	- Professionals	x	x	x
	SDQ	- Professionals - Children			
Behavioral & Emotional Factors	Youth Self-Report (YSR)	- Children and adolescents from the age of 11–21	x	x	x
Well-being	WHO-5	- Children and adolescents from the age of 11–21 - professionals	x	x	x
Loneliness	from WHO-HBSC-Survey	- Children and adolescents from the age of 11–21	x	x	x
State Self Esteem	SSES-6	- Children and adolescents from the age of 11–21	x	x	x
Self-Efficacy	ASKU	- Children and adolescents from the age of 11–21	x	x	x
Participation & satisfaction within institution	Custom items based on CAP-DMQ, adapted items from German and Swiss studies [22,23]	- Children and adolescents from the age of 11–21	x	x	x
Relationship with case workers and other kids in the institution	Adapted questions from WHO-HBSC-Survey	Children and adolescents from the age of 11–21	x	x	
Mobile phone usage & sport	Custom items	- Children and adolescents from the age of 11–21	x	x	
Circumstances of the child’s departure	Custom items	- Professionals - Parents			x
Assessment of the support	Custom items based on Kapella [21]	- Children and adolescents from the age of 11–21			x
Satisfaction with their time in residential care	Custom items based on Kapella [24]	- Children and adolescents from the age of 11–21			x
Current living context	PFS-2	- Parents	x	x	
Contact with child	Custom items	- Parents	x	x	x
Cooperation with child and youth welfare services	PFS-2	- Parents	x	x	
Perception on provided support	Custom items	- Parents	x	x	x
Satisfaction with the residential care & child and youth service	Custom items based on Kapella [21]	- Parents			x

**Note:** T0 = admission to care, T1=during stay, *multiple times, depending on length of stay, T2 = exit from care. CBCL = Child Behavior Checklist [25]; YSR/11–18 = Youth Self-Report for ages 11–18 [25]; SDQ = strengths and difficulties questionnaire [26]; WHO-5 = Well-Being Index [27]; SSES-6 = The Six-Item State Self Esteem Scale [28]; ASKU = Short Form of the Self-Efficacy Scale [29]; CAP-DMC = Child and Adolescent Participation in Decision-Making Questionnaire; HBSC = Health Behaviour in School-aged Children [30]; PFS-2 = Protective Factors Survey, 2nd Edition [31].

#### Professional caregiver-surveys.

The survey administered to professional caregivers, who are responsible for daily care, supervision, and support of children and adolescents in out-of-home settings comprises two main parts: 1) Questions about ‘You and Your Work’ and 2) Questions about ‘Your Client’. Depending on the specific assessment time point, completion takes up to approximately 35 minutes.

Part 1 includes three sections. The first section focuses on demographic and professional background information, including participants’ age, gender, educational background, years of professional experience (both overall and at the current institution), additional qualifications, extent of employment, and supplementary roles within the child and youth welfare system. The second section addresses aspects of the professionals’ current work situation, such as job satisfaction, the number of children and adolescents in their care, perceptions of adequate staffing and scheduling, access to vacation and compensatory time, and discrepancies between contracted and actual working hours. The third section assesses the well-being of the professionals using the WHO-5 Well-Being Index, a widely used self-report measure developed by the World Health Organization to assess subjective psychological well-being [27]. The WHO-5 consists of five positively worded items measuring mood, vitality, and interest over the past two weeks on a Likert scale and serves as a screening tool for depression and general well-being.

Part 2 consists of a single section focusing on information about an individual client, who has agreed to participate in the study. This includes case-specific data such as a pseudonymous case-ID, that will be used to link data across multiple time points and participants without compromising data security, date of admission to the facility, first out-of-home placement, number of previous residential placements, source of referral, and context of the report. Additionally, it captures any prior contact between the family and child and youth welfare services, the legal framework governing the placement, reasons for child welfare support, therapeutic interventions received, and the nature and extent of contact with the client and their family. Family characteristics, regarding cooperation and engagement between family and professional caregiver are assessed using selected and adapted items from Kapella et al. [21]. Emotional and behavioral functioning of the client is measured using the Child Behavior Checklist (CBCL) [25] and Strengths and Difficulties Questionnaire (SDQ) [26]. The CBCL, part of the Achenbach System of Empirically Based Assessment (ASEBA), is a standardized tool widely used to assess internalizing and externalizing problems, as well as social functioning in children and adolescents, based on caregiver or professional reports. For technical reasons (the original version could not be displayed in the app), we made minor modifications to the social competence scale in the questionnaire. Rather than requesting separate assessments of the child’s time spent and skill level for each listed sport, activity, or organization, participants are now asked to provide an overall evaluation for each group. The CBCL is, however, an extensive questionnaire and to therefore to avoid drop-outs in the future the SDQ will also be used as a shorter measurement instrument of emotional and behavioral problems. The German version of the SDQ has shown strong comparability with the CBCL [26].

When the survey is administered at the time of a client’s exit from the facility, additional items are included to document the circumstances of the child’s departure, including the reason for exit, how well they think the child is prepared for the period after leaving residential care and any planned follow-up arrangements.

#### Child and adolescent-surveys.

The child and adolescent-survey takes up to around 30 minutes to complete, depending on the assessment time point. It is divided into two parts.

The first section is administered to all children aged 11 years and older. It begins with demographic questions, including a pseudonymous case ID, age, sex, place of birth, migration background, and first language. Following the demographic items, a number of standardized instruments and custom-developed questions are used to assess psychological and behavioral characteristics.

Self-esteem will be assessed using The Six-Item State Self-Esteem Scale (SSES-6) [28] based on the German translation of SSES-R by Rudolph et al. [32,33]. Although the instrument was originally developed for use in adults, the items are linguistically simple and therefore considered appropriate for children from the age of 11 years.

Self-efficacy is assessed using the Short Form of the Self-Efficacy Scale [29]. This scale evaluates individuals’ beliefs in their ability to successfully perform tasks and handle various situations. Although originally validated for adults, it will be applied to children in this study due to its brief format and relevance to everyday functioning. The items have been linguistically and conceptually reviewed for appropriateness with the younger population.

Participants’ experience of loneliness is measured using a single item derived from the WHO- HBSC survey an international research collaboration conducted across Europe and North America [30].

In addition to standardized instruments, two custom-developed items are included to gather information on children’s mobile phone use and participation in sports activities.

Children’s involvement in decision-making processes is assessed using a set of adapted items, as no standardized instrument currently exists to capture this specific aspect. Two single adapted items from the Child and Adolescent Participation in Decision-Making Questionnaire (CAP-DMQ) [34], that were translated from the original English version into German using the forward-backward translation method [35] to ensure linguistic accuracy and cultural relevance are used. In addition, we adapted questions from a questionnaire conducted in Germany, as part of a study on youth welfare, and social change [23] as well as items from a satisfaction questionnaire by Equals on results-oriented quality assurance in social education institutions in Switzerland [22] and from a questionnaire of a conducted evaluation of the Federal Child and Youth Welfare Act in Austria [24].

Additionally, the survey includes items on children’s relationships with professionals and peer relationships within the residential facility, based on constructs from the WHO-HBSC survey [24,30]. These questions focus on the quality of friendships and family relationships as perceived by the children.

Part Two is called “Questions About Your Health and Behavior”. It includes the youth self-report counterparts to the CBCL and SDQ (Youth Self-Report for ages 11–18 (YSR/11–18)/SDQ) [25,26]. These instruments allow adolescents to report on their own emotional and behavioral functioning. Similarly to the professional’s survey, we made minor modifications to the social competence scale in the questionnaire. Rather than requesting separate assessments of the child’s time spent and skill level for each listed sport, activity, or organization, participants are now asked to provide an overall evaluation for each group.

The final survey, conducted at the time of exit from residential care, includes custom items on the type of accommodation planned after leaving care, how well they feel prepared for the period after leaving residential care, as well as questions about the child’s satisfaction with their time in residential care. These are followed by a set of items taken directly from the evaluation of child and youth care services in the Austrian state of Vorarlberg conducted by Kapella et al. [21], focusing on the child’s assessment of the support received during their placement.

#### Parent-surveys.

The parent-survey takes up to 15 minutes to complete and is divided into two main parts: Part One: ‘Questions About You and Your Current Living Situation’, and Part Two: ‘Questions About Your Child in Residential Care’.

Part 1 collects demographic information, including the respondent’s year of birth, sex, highest level of education, migration background, current employment status, family structure, and living situation (e.g., relationship status, household composition). It also includes questions about the respondent’s past experiences with child and youth welfare services.

In addition, this section assesses the current living context, with a focus on perceived social support and financial stability. These items are drawn from the Protective Factors Survey, 2nd Edition (PFS-2) a tool developed for use with parents and caregivers involved in family support and child maltreatment prevention services in the US [31]. The PFS-2 items were translated into German using the forward-backward translation method [35] to ensure linguistic and conceptual equivalence.

Part 2 includes case-specific information such as the pseudonym case ID, the respondent’s relationship to the participating child, and the form and frequency of contact with the child. It also includes questions on cooperation with child and youth welfare service, drawn from the PFS-2 [31], along with two custom-developed items.

Furthermore, this section contains additional custom items assessing how helpful the respondent perceives the support provided through residential care to be—both for themselves and for their family as a whole.

The final survey administered at the time of a child’s exit from residential care includes a custom item assessing the type of accommodation planned following their child’s departure. It also contains questions addressing the parental guardian’s satisfaction with the residential care experience and the child and youth welfare service more broadly, as well as any perceived areas for improvement based on the evaluation study of Kapella et al. [21].

#### Statistical analysis.

The quantitative data analysis will comprise both descriptive and inferential statistical methods. Analyses will be conducted separately for the three target groups (professionals, children and adolescents, parents/guardians) and integrated where appropriate to explore associations across perspectives. Quantitative analyses will be conducted using the statistical software programs SPSS (Version 27 or later) [36] and R (Version 4.3 or later) [37]. Qualitative data from open questions will be evaluated using the software MAXQDA [36].

*(1) Professional Background and Working Conditions*

We will conduct descriptive analyses of the professionals’ background data, including education, work experience, employment type (e.g., full-time, part-time), and perceived workload or staff-to-child ratio at their institution.

*(2) Well-being and Job Satisfaction*

Data from standardized satisfaction and well-being questionnaires for professionals will be analyzed to explore levels of job satisfaction, psychological well-being, and perceived institutional support. Group differences (e.g., by experience level or institutional size) will be examined using ANOVA and regression analyses.

*(3) Child-Specific Data*

We will analyze data reported by children, as well as reports from professionals. Variables include emotional and behavioral functioning (CBCL/SDQ and YSR/11–18R), self-esteem, self-efficacy, well-being, perceived participation and satisfaction within institution, loneliness and smartphone usage and sport. These will be examined both cross-sectionally and, in later waves, longitudinally to identify changes over time. Group comparisons will be conducted based on demographic characteristics (e.g., age, sex, migration background) as well as child welfare-specific factors (e.g., reasons for out-of-home care or care history) and family- or institution-level variables using regression models.

*(4) Family Situation and Parental Perspective*

Sociodemographic data of parents (e.g., education, employment status, household composition) will be analyzed descriptively and included as covariates in further analyses. Parental responses regarding the current family situation, perceived collaboration with care institutions, and perceived support from the child welfare system will be described and examined in relation to child outcomes and institutional characteristics using ANOVA and regression analysis.

*(5) Interrelations Between Variables*

Associations between the key constructs from all three target groups (e.g., professional working conditions, child well-being, family situation) will be analyzed using correlation matrices, multiple regression models, and—where appropriate—structural equation modeling (SEM) to identify potential pathways or mediating effects.

*(6) Handling of Longitudinal Data*

For institutions participating in multiple data waves, repeated measures analyses (e.g., mixed-effects models) will be used to explore change over time and predictors of change in children’s well-being and participation. Missing data will be handled using appropriate imputation methods or sensitivity analyses, depending on the extent and pattern of missingness.

*(7) Inclusion of Open-Ended Responses in Quantitative Analyses*

Open-ended survey responses will be analyzed using qualitative content analysis. Based on an inductive coding process, answers will be grouped into thematic categories. These categories will then be quantified (e.g., through frequency coding or binary indicators) and integrated into the quantitative dataset systematically using qualitative data analysis software MAXQDA [38] to ensure transparency and traceability. To enhance reliability, a subset of responses will be double coded by an additional researcher, and any discrepancies will be discussed until consensus is reached.

### Accompanying qualitative approach

In order to supplement the quantitative findings and gain a richer understanding of the residential out-of-home care landscape in Lower Austria, an in-depth qualitative part of the study is planned. This qualitative phase follows the distribution of a quantitative survey and is designed to explore the everyday practices, challenges, lived experience and contextual factors influencing child development within residential care settings. In addition, qualitative methods make it possible to respond to potential accessibility challenges during quantitative surveys, which are to be expected in the group of parents in particular, but can also occur among children. This procedure ensures that the study captures a diverse range of perspectives, even if the initially planned surveys do not achieve an adequate response rate among all target groups.

The specific qualitative methods and the selection of participants are developed based on theoretical sampling of grounded theory methodology [39] in line with the results of the quantitative surveys, and existing circumstances in the research field. For example, qualitative expert interviews or focus groups will target professionals who are directly involved in out-of-home care placements, including caregivers, social workers, and administrative staff. Family discussions can also be considered as another effective method for identifying specific dynamics in out-of-home care placements or family structures. Different modified qualitative interview techniques with children, or adolescents, and their parents can also be conducted, depending on their availability and the quantitative results we already receive [40]. Recruitment will occur through the “Dachverband Österreichischer Kinder- und Jugendhilfe,” the Austrian umbrella organization for child and youth welfare services. Participation will be entirely voluntary, with informed consent obtained from all participants prior to data collection.

#### Qualitative methodological overview.

The qualitative part of the study will follow a semi-structured format, allowing both structure and consistency to compare cases, as well as flexibility to explore individual perspectives in depth. Key thematic areas will include the structural and procedural organization of care facilities, the allocation and adequacy of resources for child support, and the identification of facilitators and barriers and their arrangements to child development. These factors will be examined across multiple dimensions—organizational, procedural, client-specific, and personal. Whereby connections, and the interplay between these multiple dimensions are also examined, and their everyday practical forms are considered.

Qualitative data will be audio-recorded (with permission) and transcribed verbatim with the software f4analyse [41]. The transcripts will be analyzed thematically, following qualitative content coding techniques [42] to identify, compare, and refine categories emerging from the data. Data management and coding will be conducted with MAXQDA software [38] to facilitate systematic analysis and traceability. This qualitative inquiry aims to generate an in-depth understanding of the systemic and individual-level mechanisms influencing outcomes for children in residential care, thereby contributing to evidence-informed policy development and practice enhancement in the region.

### Communicative validation

In order to provide feedback on the research process from the subjects of research as a quality criterion for the findings, communicative validation is carried out alongside the study. Research questions, instruments, methods, and subsequently the results are negotiated communicatively with relevant stakeholders and experts from the field of research throughout the research process. In addition, negotiations are taking place regarding the various roles that all participants will assume within the research activities. The accompanying communicative validation enables all participants and their articulated and explained needs, stances, and desires to be taken into account in the context of their positions [43].

During the planning phase of the study, 18 coordination meetings were held with representatives from the administration and experts from the field. In these meetings, the research design and survey instruments were jointly developed and critically discussed to integrate scientific conceptualization with practical expertise and field experience.

Further pre-tests were conducted with the target groups before the start of the study to assess the comprehensibility and suitability of the survey instruments. This process can also be understood as a step toward communicative validation of the research design.

In future, results from the long term-study will be reflected and discussed once a year with stakeholders and professionals from the field of child and youth welfare services, and their views on the validity of the results will be considered. Officials and other experts can participate in the research process and contribute their analyses and interpretations of the results. This process of Communicative Validation ensures that the results are relevant to the practical work of child and youth services, and that the results are prepared and presented in an appropriate manner. It also enables further development and quality improvement of child and youth welfare services.

### Ethical considerations

The studies were approved by Ethics Committee REMOVED FOR PEER REVIEW. For each of the participating groups we have developed specific information on the study, our data protection policy and informed consent, which will be provided via ESMira App [15], Limesurvey [16], and as printed information. The participants will give their informed consent by ticking the digital consent box in ESMira, and Limesurvey [16].

In line with the EU General Data Protection Regulation, the university data protection policies and national regulations, informed consent is required from a guardian for children under the age of 14. In the case of children in out-of-home placements, the professional care givers (social workers, social pedagogues) are legally permitted to consent on their behalf. Adolescents aged 14 and above may participate independently. However, as we also ask care givers to provide information about the adolescent in our questionnaire, we ask them (in addition to their own consent) to ensure the adolescents permission; before ESMira, and Limesurvey presents the questions regarding the adolescent to a participating care giver, they have to confirm the adolescents’ consent. Without this confirmation, the survey subsequently ends with an information on our data protection policy. Participation in the study is entirely voluntary and pseudonymous, and all individual responses will be treated as strictly confidential. Through the ESMira App, and Limesurvey, all participants have their data protection information including the terms and conditions of the study participation constantly available, as well as the possibility to tick the box to withdraw from the study at any point in time. In the case of withdrawals, all data collected up to that point will be deleted from the data set of the study and excluded from further analyses.

To maintain participant confidentiality while enabling linkage across data sources (e.g., perspectives of professionals, children, and guardians) and multiple time points, participants will generate a pseudonymous ID code when completing the online survey. In coordination with the university’s data protection office, the ID code is created using the first letters of the participant’s (child or professional) first and last names, the day of birth, and the last two digits of the birth year. The ID code also serves to double check the consent confirmation of adolescents and care providers.

Interview transcripts will be pseudonymized by removing identifying details before analysis.

Given the vulnerability of children in residential care, they are informed that they can seek help from their guardians or contact the research team via an anonymous chat feature integrated into the survey app.

### Data management

This longitudinal mixed-methods study involves both quantitative and qualitative data collection. Quantitative data will be gathered through an online survey using the ESMira App [15], and Limesurvey [16], while qualitative data will be obtained through audio-recorded interviews and subsequently transcribed. All data will be securely stored on encrypted servers at the University for Continuing Education Krems compliant with university IT security guidelines. Access to the data is strictly limited to members of the research team. The regional child and youth welfare service will not have access to any individual-level data. Aggregated and anonymized data will be published open access in the DOOR repository of the University for Continuing Education Krems alongside each relevant summary report or publication.

## Discussion

### Potential impact and innovation

To date, no comparable longitudinal – mixed method study is known to exist in Austria that systematically examines the conditions under which children and their families become recipients of full-time residential care services in Lower Austria, or how children develop over time while placed in such care from multiple perspectives. This study will provide valuable insights into the trajectories of children in residential care and the contextual factors that shape their experiences and outcomes. The findings have the potential to inform practice by supporting evidence-based decision-making processes and highlighting areas where intervention may be most effective. Furthermore, the study can serve as a foundation for more detailed, future research on key factors influencing child development in out-of-home care settings.

One important innovation of this project is the use of an open-ended survey design that could lead to the development of a standardized documentation tool for the child and youth welfare system in Lower Austria. Such a tool could be implemented more broadly when children enter residential care, allowing for more consistent data collection across institutions. This would not only facilitate future scientific research but also play a significant role in quality monitoring and assurance across the child welfare system.

### Limitations and challenges

Given the complexity of the field of child welfare and residential care, not all relevant aspects could be included in the current study design. In collaboration with practitioners, we carefully prioritized and selected the most critical factors for inclusion. Nevertheless, the study may not capture the full range of experiences and variables influencing outcomes in residential care.

Recruiting participants presents another major challenge. The study depends on the support and cooperation of child and youth welfare organizations in Lower Austria. A key success factor will be whether professionals in these organizations can allocate time within their busy schedules to participate and actively recruit children for the study. Engaging with parents is likely to be particularly difficult. If it becomes evident that professionals are unable to involve parents directly, we may need to collaborate with social workers who maintain closer contact with families to facilitate this aspect of recruitment.

Additionally, repeated survey participation may result in participation fatigue among respondents. This concern is especially relevant for children, who may find the repeated completion of questionnaires demanding, and for professionals who are supporting multiple children enrolled in the study. To mitigate this risk, each survey is divided into two parts, which can be completed on separate days. Each part includes an introductory note indicating the estimated time required to complete it, helping participants plan accordingly and reducing burden.
